# Changing survival, memory cell compartment, and T-helper balance of lymphocytes between severe and mild asthma

**DOI:** 10.1186/1471-2172-9-73

**Published:** 2008-12-16

**Authors:** AS Abdulamir, RR Hafidh, F Abubakar, KA Abbas

**Affiliations:** 1 Microbiology Research Department, University Putra Malaysia, 43400, UPM, Serdang, Malaysia; 2 Institute of Bioscience, University Putra Malaysia, 43400, UPM, Serdang, Selangor Darul Ehsan, Malaysia; 3 Faculty of Food Science and Technology, University Putra Malaysia, 43400, UPM, Serdang, Malaysia

## Abstract

**Background:**

Asthma is a complicated network of inflammatory reactions. It is classified into mild, moderate, and severe persistent asthma. The success of asthma therapy relies much on understanding the underlying mechanisms of inflammation at each stage of asthma severity. The aim of this study was to explore the differences in apoptotic potential, CD4/CD8 ratio, memory compartment, and T- helper (Th) 1 and 2 profile of peripheral blood lymphocytes (PBL) in patients with mild intermittent asthma and severe persistent asthma during exacerbation periods.

**Results:**

Four research lines were investigated and compared among mild asthmatics, severe asthmatics, and healthy groups by applying immunocytochemical staining of PBL. Antiapoptotic and proapoptotic proteins with Bcl-2/Bax ratio, CD4, CD8 markers with CD4^+^/CD8^+ ^ratio, CD45RO^+^, CD45RA^+ ^markers with memory/naïve ratio (CD45RO^+^/CD45RA^+^). Th2/Th1 cytokines balance represented by IL-4/IFN-γ ratio was measured by enzyme-linked immunosorbent assay (ELISA) for in vitro PBL cytokine synthesis. It was found that Bcl-2/Bax ratio was higher in severe than in mild asthmatics which in turn was higher than in healthy group. And memory/naïve ratio of PBL was higher in severe than in mild asthmatics. Moreover, memory cells, CD45RO^+ ^and CD45RO^+^/CD45RA^+ ^ratio were correlated directly with Bcl-2/Bax, in severe and mild asthma patients. In contrast, CD4^+^/CD8^+ ^ratio was not changed significantly among healthy group, mild and severe asthmatics. However, CD8^+ ^cells were correlated directly with memory cells, CD45RO^+^, in severe asthmatics only. Interestingly, the dominant profile of cytokines appeared to change from T helper 2 (Th2) in mild asthmatics to T helper 1 (Th1) in severe asthmatics where the lowest in vitro IL-4/IFN-γ ratio and highest IFN-γ were found.

**Conclusion:**

It was concluded that the underlying mechanisms of inflammation might vary greatly with asthma stage of severity. Mild intermittent asthma is mainly Th2 allergen-oriented reaction during exacerbations with good level of apoptosis making the inflammation as self-limiting, while in severe persistent asthma, the inflammatory reaction mediated mainly by Th1 cytokines with progressive loss of apoptosis leading to longer exacerbations, largely expanded memory cells, CD45RO^+^, leading to persistent baseline inflammation.

## Background

Inflammation is the central feature of asthma pathogenesis and the core of its clinical manifestations that plays the main role in airway obstruction and hyperresponsiveness [1]. Asthma is a waxing and waning disease that leads to structural changes in the airways and it is a common disorder in which chronic inflammation of the bronchial tubes makes them swell and cause narrowing of the airways [2]. According to the United States National Heart, Lung and Blood Institute, asthma is classified into mild intermittent, mild persistent, moderate persistent, and severe persistent [3]. Apoptosis malfunctions have been involved in the chronic status of many inflammatory diseases [4] including asthma leading to long lasting unwanted inflammation and adverse effects of immune cells recruitment and activity [5]. It was found that there is a significant increase in percentage of anti-apoptotic Bcl-2 protein in asthmatic patients, and a decrease in the percentage of pro-apoptotic Bax protein in patients compared with healthy individuals [6].

The 'Th2 hypothesis for asthma' was first suggested by Mosmann in 1989 [7], who had earlier discovered the presence of two distinct subtypes of helper T cells in mice, namely, T helper 1 (Th1) and T helper 2 (Th2) [8]. These two subclasses of T helper cells differ in their production of cytokines which cross-inhibit each other. IFN-γ amplifies Th1-cytokines profile [9], therefore, IFN-γ is considered as the prototype cytokine of Th1 lymphocytes which cross regulates IL-4, the prototype cytokine of Th2 lymphocytes in asthma [10]. In fact, asthma has long been considered as a purely Th2 disease [7,8], however a report [11] stated that both Th2 and Th1 chemokines may be involved in allergen-induced airway inflammation. Moreover, a study [12] revealed that some of the Th1-related chemokines were predominant during human allergic pulmonary late-phase reaction.

Regarding CD4 and CD8 markers, the role of CD4^+ ^T cells in the immunopathogenesis of asthma is well documented, though little is known about the role of CD8^+ ^T cells. A study [13] demonstrated that allergic asthma in children showed that CD8^+ ^T cells might play a role in the pathogenesis of asthma. Regarding the effector memory cells of PBL, it has been shown that CD45RO^+^, may provide pro-inflammatory signals that contribute to the persistent airway inflammation of asthma [14]. And it was also showed that it is not clear whether the memory compartment of T cells, CD45RO^+ ^cells, vary with the progression of asthma disease or not [13].

There is to date no or very few published studies demonstrating the relationship of changed Th1/Th2 balance with changes of apoptosis, changes of memory cell compartment, and changes of CD4^+^/CD8^+ ^ratio in peripheral blood lymphocytes (PBL) between mild intermittent and severe persistent asthmatic adult patients during exacerbations. The previous studies either were conducted on asthmatic children or involved only part of the research lines implemented in this study. Hence, this study was designed to explore the correlations and changes in Th1/Th2 balance, represented by PBL in vitro synthesis of IL-4 and IFN-γ and their ratio, PBL CD4^+^/CD8^+ ^ratio, PBL Bcl-2/Bax ratio, and PBL memory/naïve CD45RO^+^/CD45RA^+ ^ratio in healthy volunteers, mild intermittent, and severe persistent asthmatic patients. This exploration is believed to be essential for acquiring a better understanding for the dynamic nature of immune reaction and inflammatory process that take place in different types and stages of asthma. This could help physicians employ more adjustable treatments on asthma targeting the real causative factors specifically.

## Methods

### Study population

Fifty two asthmatic patients were involved in this study during the period from February 2007 to March 2008 in Selangor, Malaysia. The age of the studied patients ranged from 17 to 53 years, none of them was regular smoker or has other major illnesses. Thirty six patients were selected with mild intermittent asthma and 16 with severe persistent asthma. All patients were diagnosed by a specialist of respiratory diseases and diagnosis was confirmed by pulmonary function tests in the central Kuala Lumpur hospital. Twenty age- and sex-matched healthy volunteers (HV) were involved in this study, aged 23 to 55 years. HV had no history of smoking or any medical disease, and their chest radiograms and pulmonary function tests showed no evidence of respiratory diseases. Five mL of blood were withdrawn from asthmatic patients and HV for PBL isolation. Blood was sampled from asthmatic patients within the first 12 hours of asthma exacerbation. Moreover, all involved patients were subjected to similar modes of therapy to avoid discrepancies in therapy protocols and to equalize the interfering effects of the oral or inhaled therapy with the study findings. Written consents were granted by all patients and HV for interviewing and blood sampling. Dealing with human subjects was carried out within the scope of Helsinki declaration of ethical principles of medical research and permission was granted from the ethics committee of biomedical research of University Putra Malaysia.

### Antibodies

Mouse monoclonal antibodies for immunocytochemistry (ICC) assay were derived from (Abcam plc, UK) and as follows; anti-human CD4 antibody (B379), anti-human CD8 (BL-Ts 8/2), anti-human CD45RO (2Q1392), anti-human CD45RA (MEM-56), anti-human Bax (ID3), and anti-human Bcl-2 (4D7). For ELISA, mouse monoclonal antibodies were used as follows; anti-human IL-4 (NYRhIL4) from (Abcam plc, UK) and anti-human IFN-γ (25718) from (Sigma, USA). Abcam Biotinylated goat secondary antibodies (ab6785) were used against mouse immunoglobulins in both ELISA and ICC assays.

### Isolation of peripheral blood lymphocytes

PBL were isolated from heparinized whole blood of asthmatic patients in order to prepare a pure population of lymphocytes. PBL separation was done in ultraviolet hood by implementing the gradient density sedimentation technique using Ficoll hypaque (Sigma). Diluted blood 1:2 in RPMI 1640 media (Sigma) was added slowly onto below Ficoll layer to create two interfacing layers. By the effect of the centrifugal force, lymphocytes were grouped into one web-like layer which then was withdrawn and washed three times with phosphate buffered saline (PBS) (Sigma). Final concentration of lymphocytes suspension was adjusted to 1 × 10^6 ^cells/mL [15].

### Immunocytochemistry for peripheral blood lymphocytes

ICC procedure was carried out according to the instructions of the manufacturer of primary and secondary antibodies (Abcam) with modifications. 200 μl of 1 × 10^6 ^cells/mL of lymphocytes suspension were aliquoted into wells of the cytospin. Cytospins were performed, 650 rpm for 6 minutes on silane-coated slides. Slides were dried in a desiccation chamber for overnight. Slides then were fixed in 2% paraformaldehyde (BDH) for 20 minutes at room temperature and washed 3 times in PBS. Slides were incubated in 95°C preheated antigen retrieval buffer, 100 mM Tris (Sigma), 5% (w/v) urea (Merck), pH 9.5 for 10 minutes in a water bath. After 3 times PBS washing, cells were permeabilized for 15 minutes at room temperature in blocking buffer, 3% BSA (Merck) in PBS, plus 0.1% Triton X-100 (Sigma) followed by blocking of nonspecific binding in blocking buffer for 1 hour at room temperature.

**Figure 1:**
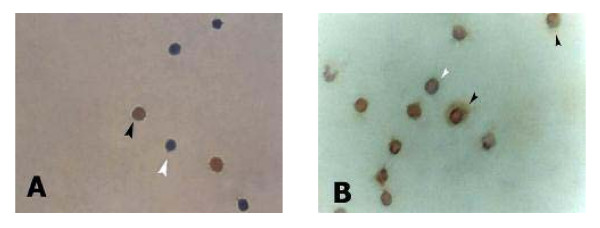
**The immunocytochemical staining of PBL with brown color DAB-chromogen**. Black arrow refers to positive cells with overall DAB dark brown staining. White arrow refers to negative cells with overall blue hematoxylin counterstaining. (A) An example of few positively immunostained cells with Bcl-2 in HV. (B) An example of numerous positively immunostained cells with Bcl-2 in severe asthmatic patients. The photos were taken at magnification power of 100×.

One hundred μl of mouse monoclonal antibodies diluted 1:50 in blocking buffer at final concentration 0.005 mg/mL were added on ICC slides against human Bax, Bcl-2, CD4, CD8, CD45RO, and CD45RA. Slides were incubated overnight in a humid chamber at 4°C. Next day, slides were rinsed gently with PBS-0.05% Tween20 (Merck) and placed in fresh PBS-Tween bath for 1 minute. 100 μl of the biotinylated goat anti-mouse secondary antibodies 1:400 diluted in PBS at final concentration 0.00125 mg/mL were added and incubated in humid chamber for 1 hour at 37°C. After washing with PBS-Tween20 solution, two drops of streptaviden-Horseradish peroxidase reagent (Dako) was applied, slides were placed in humid chamber and incubated for 30 minutes at 37°C. The prepared DAB-substrate chromogen (Dako) solution was then applied and slides were incubated in dark for 20 minutes at room temperature. Mayer's hematoxylin stain was used as counterstain, and slides were then dehydrated and mounted with DPX mounting fluid. The immunostained cells at moderate to intense dark brown color were considered positive and other cells were considered negative as seen in (Fig. 1). Under light microscope, the number of brown stained cells out of 200 countable total cells was calculated to measure the percentage of the positive PBL expressing Bax, Bcl-2, CD4, CD8, CD45RO, and CD45RA.

### Enzyme-linked immunosorbent assay for in vitro synthesis of PBL cytokines

PBL cells were incubated for 3 days at 37°C in 1.5 mL microcentrifuge tubes containing complete RPMI-1640 medium (Sigma) with 200 U/mL penicillin G, 200 μg/mL streptomycin, and 10% human AB serum at a concentration of 1 × 10^6 ^cells/mL. At 4th day, PBL cells were centrifuged at 2000 g for 5–10 minutes, and then the supernatant was collected in a sterile tube. The collected supernatant of the in vitro PBL culture was diluted 1:10 in carbonate-bicarbonate coating buffer (1.59 g/L carbonate & 2.93 g/L bicarbonate) (Merck). 50 μl/well of the supernatant-coating buffer mixture were added in duplicates to a sterile 96 wells microtiter plate which was incubated overnight at 4°C. Next day, the remaining fluid was aspirated and wells were washed twice with washing buffer (PBS, 1% BSA w/v from Merck, 0.05% v/v Tween 20 from Merck). Blocking buffer (PBS, 1% BSA w/v) was then added for 1 hour. After a washing step, 50 μl of mouse monoclonal antibodies diluted 1:50 in blocking buffer at final concentration 0.002 mg/mL were added and incubated for 2 hours at 37°C. After a washing step, 50 ul of the biotinylated goat anti-mouse antibodies at final concentration 0.003 mg/mL were added and incubated for 1 hour at 37°C. After washing, 50 ul of streptaviden-horseradish peroxidase reagent (Dako) were added and incubated for 30 minutes at 37°C. After washing, 50 ul of OPD-H_2_O_2 _chromogen solution (Sigma) were added and microtiter plate was kept in dark for 15 minutes at room temperature. ELISA readings for optical density (OD) were measured immediately by microtiter reader A-1764A (Beckman) at wavelength of 492 nm [16].

### Statistical analysis

The statistical analysis was preformed using software SPSS version 10 and MS Excel 2000. After confirming the normal distribution pattern of both ICC and ELISA values by using normal distribution tests, mean ± 2 standard error and parametric multivariate student t-tests were used to evaluate the significance of differences. Pearson's correlation coefficient was used for correlating the level of expression of the studied targets in the involved groups of asthmatic patients.

## Results

### The immunostaining of PBL for Bax, Bcl-2, CD4, CD8, CD45RO, and CD45RA

**Figure 2:**
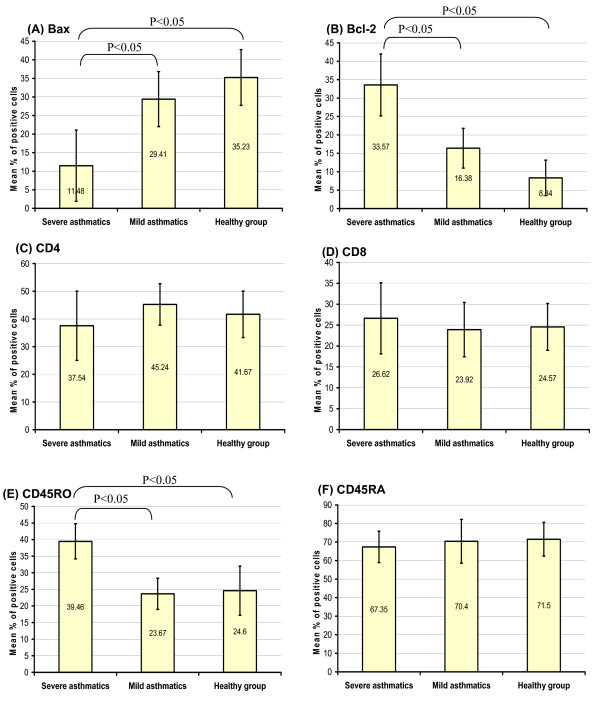
**The mean percentages ± 2 standard errors of the ICC positively stained PBL for the studied markers in severe asthmatic patients, mild asthmatic patients, and HV group**. (A) Bax was significantly lower in severe asthmatics than other groups (B) Bcl-2 was significantly higher in severe asthmatics than other groups. (C) No significant difference in CD4 expression among the studied groups. (D) No significant difference in CD8 expression among the studied groups. (E) CD45RO was significantly higher in severe asthmatics than other groups. (F) No significant difference in CD45RA expression among the studied groups.

**Figure 3:**
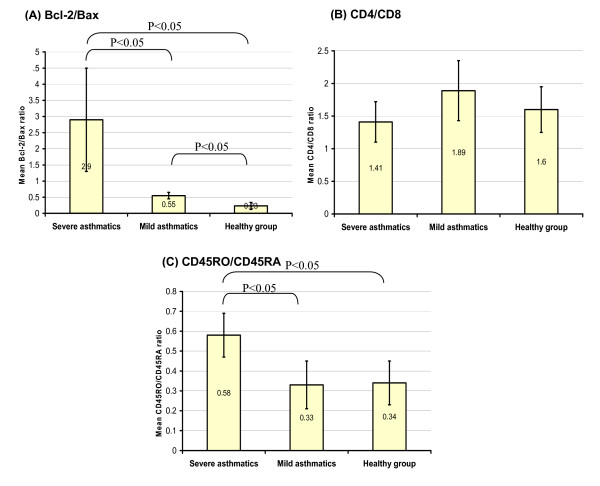
**The mean ratio ± 2 standard errors of Bcl-2/Bax, CD4/CD8, and CD45RO/CD45RA**. (A) BCL-2/Bax ratio was significantly higher in severe than in mild asthmatics which in turn was higher than in HV group. (B) The difference of mean CD4^+^/CD8^+ ^ratio in severe asthmatics, mild asthmatics, and HV was statistically insignificant. (C) The mean CD45RO^+^/CD45RA^+ ^ratio was significantly higher in severe asthmatics than other groups.

The isolated PBL from mild asthmatics, severe asthmatics, and HV groups were examined by immunocytochemistry. It was found that the mean percentage of the positive PBL for Bax was lower in severe asthmatics, 11.48 ± 9.6, than the close figures found in both mild, 29.41 ± 7.4, and HV, 35.23 ± 7.5 (P < 0.05). In contrast, the mean percentage of positive PBL for Bcl-2 was higher in severe asthmatics, 33.57 ± 8.4, than the close figures in both mild asthmatics, 16.38 ± 5.4, and HV, 8.34 ± 4.8 (P < 0.05). Accordingly, the mean Bcl-2/Bax ratio was calculated and was at highest level in the severe asthmatic patients, 2.9 ± 1.6, and significantly higher than in mild asthmatics, 0.55 ± 0.1, (P < 0.05) and in mild asthmatics was in turn higher than in HV, 0.23 ± 0.11, (P < 0.05) (Fig. 2 and 3).

It was found that the mean percentage of CD4^+ ^PBL was not significantly different in mild asthmatics, 45.24 ± 7.5, in HV, 41.67 ± 8.4, and in severe asthmatics, 37.54 ± 12.5 (P > 0.05). Similarly, the mean percentage of CD8^+ ^PBL was not significantly different in severe asthmatics, 26.62 ± 8.5, mild asthmatics, 23.92 ± 6.5, and HV, 24.57 ± 5.6 (P > 0.05). Accordingly, the mean CD4^+^/CD8^+ ^ratio was not significantly different too in severe asthmatics, 1.41 ± 0.31, mild asthmatics, 1.89 ± 0.46, and 1.6 ± 0.35 (P > 0.05) (Fig. 2 and 3).

Regarding CD45RO and CD45RA markers, it was found that the mean percentage of the memory CD45RO^+ ^PBL was higher in severe asthmatics, 39.46 ± 5.3 than in mild asthmatics, 23.67 ± 4.7 and HV 24.6 ± 7.4 (P < 0.05), while no significant difference was found for the mean percentage of naïve CD45RA^+ ^PBL in severe asthmatics, 67.35 ± 8.5, mild asthmatics, 70.4 ± 11.8, and HV, 71.5 ± 9.1 (P > 0.05). Accordingly, the memory/naïve CD45RO^+^/CD45RA^+ ^ratio in severe asthmatics, 0.58 ± 0.11, was higher than that in mild asthmatics, 0.33 ± 0.12, and in HV group, 0.34 ± 0.11 (P < 0.05) (Fig. 2 and 3).

### Enzyme-linked immunosorbent assay for IL-4 and IFN-γ

**Figure 4:**
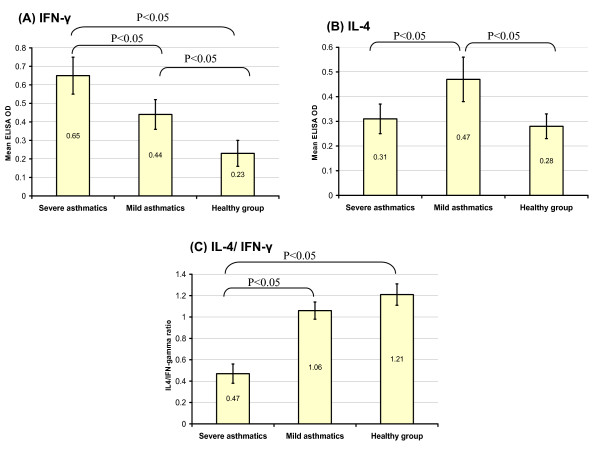
**(A) The mean OD value ± 2 standard errors of in vitro PBL synthesis of IFN-γ increased significantly with asthma severity**. (B) The mean OD value ± 2 standard errors of in vitro PBL synthesis of IL-4 was significantly highest in mild asthmatics than severe asthmatics and HV groups. (C) The mean ratio ± 2 standard errors of IL-4/IFN-γ was remarkably lower in severe asthmatics than mild asthmatics and HV groups.

The isolated PBL from severe and mild asthmatics as well as HV group were cultured in vitro in complete RPMI medium for 3 days and the level of the in vitro synthesis of IL-4 and IFN-γ was measured by ELISA in terms of optical density (OD) values. It was found that the in vitro synthesis of IFN-γ was increasing in parallel with disease severity. It was found that OD level of IFN-γ in severe asthmatics, 0.65 ± 0.1, was significantly higher than in mild asthmatics, 0.44 ± 0.08 (P < 0.05) which in turn was higher than in HV, 0.23 ± 0.07 (P < 0.05) (Fig. 4). On the other hand, OD level of IL-4 was significantly highest in mild asthmatics, 0.47 ± 0.09, in comparison with severe asthmatics, 0.31 ± 0.06 and HV, 0.28 ± 0.05 (P < 0.05) (Fig. 4). Accordingly, the IL-4/IFN-γ ratio was calculated giving a clue on the polarization of the isolated PBL. The lower IL-4/IFN-γ ratio, the more Th1 polarized PBL and the higher IL-4/IFN-γ ratio, the more Th2 polarized PBL. IL-4/IFN-γ ratio was remarkably lower in severe asthmatics, 0.47 ± 0.09, than in HV, 1.21 ± 0.1, and mild asthmatics, 1.06 ± 0.08 (P < 0.05) (Fig. 4).

### The correlations in the studied markers and cytokines

The Pearson's correlation coefficient (*r*) was used as a tool to shed light on the correlating behavior of the expressed markers and the synthesized cytokines of PBL with each other. It was found that the PBL expression of Bax was inversely correlated with Bcl-2, in mild asthmatics, *r *= -0.65, severe asthmatics, *r *= -0.43 and in HV, *r *= -0.47 groups (P < 0.05). CD45RO^+^/CD45RA^+ ^ratio was correlated directly with Bcl-2/Bax ratio, *r *= +0.53, in severe asthmatics only (P < 0.05). The level of CD45RO expression was positively correlated with Bcl-2 in PBL of severe asthmatics, *r *= +0.74, and mild asthmatics, *r *= +0.63 (P < 0.05). The level of CD8 expression was positively correlated with that of CD45RO, *r *= +0.62 in severe asthmatics only (P < 0.05).

## Discussion

In this study, ICC was used to visualize the expression of the studied markers. Although ICC is not a sophisticated procedure, it is considered by some reports as more sensitive and specific than flow cytometry [17]. Moreover, ICC has long been the golden standard for evaluating flow cytometry and devoid of the flow cytometry's major drawback which is the lack of visual control and morphology of the stained targets [18]. On the other hand, non-sandwich ELISA protocol was used in this study by coating the solid phase with the supernatant of in vitro PBL culture. Even though this method is a bit less sensitive than sandwich ELISA, the art showed that blocking the non-specific epitopes of the coated antigens renders the assay more specific than sandwich ELISA which depends on the coated antibodies. Moreover, OD levels of ELISA were used directly in the analysis of the results without using calibrated concentrations because the aim of this study was to compare the expression level of the studied targets in the involved groups rather than measure the real concentrations. It was essential to use consistent protocols, conditions, and devices for ELISA to prepare the suitable background for a reliable comparative analysis of the target markers and cytokines.

It was found that the survival of PBL in asthmatic patients was higher than in HV and increases with asthma severity, represented by increasing Bcl-2/Bax ratio. The results of this study are in agreement with other recent studies revealed that Bcl-2 or Bcl-2/Bax ratio increase in asthmatic lungs in comparison with normal lungs [14,19]. In asthma, where the inflammation is the major problem, lymphocyte persistence may play a key role in the pathophysiology that lead to chronic persistent airway inflammation [20]. Since Bcl-2 and Bax were inversely correlated in asthmatic patients and HV group, this confirmed that Bcl-2 protein can inhibit the expression and effect of pro-apoptotic Bax proteins. The progressive decrease of apoptosis with the increase of severity, chronicity, and persistence of asthmatic inflammation refers to the role of increased life span of the activated PBL in the progression of the disease.

Decreased apoptosis in PBL may explain the extensive exacerbations but not the persistent inflammatory reactions seen exclusively in severe persistent asthma. Persistent inflammation needs abundant presence of readily primed memory lymphocytes in lung and peripheral blood [21]. Therefore, the memory/naïve ratio of PBL was measured to investigate the changes in memory compartment with asthma severity. The percentage of CD45RO^+ ^PBL as well as memory/naïve, CD45RO^+^/CD45RA^+^, ratio were much higher in severe asthmatics than in mild asthmatics or in HV group. This indicated that the memory compartment in PBL of severe asthmatic patients was expanded significantly and this might lead to persistent inflammatory reactions in lung with or without the presence of the specific allergen. Few studies have reported imbalance in memory CD45RO^+ ^cells in the peripheral blood of patients with asthma and allergy but the results were inconsistent [22-24]. Others revealed that an increase in the memory cells, CD54RO^+^, in lung or peripheral blood may be an evidence of chronic inflammation in asthmatic children [13]. However, they found no correlation between the severity of asthma and the expression of the memory marker, CD45RO, on CD4+ T cells. The correlation of CD45RO^+ ^PBL and CD45RO^+^/CD45RA^+ ^ratio with apoptosis markers was investigated. It was found that percentages of CD45RO^+ ^PBL and CD45RO^+^/CD45RA^+ ^ratio were positively correlated with Bcl-2 and Bcl-2/Bax ratio respectively in mild and severe asthmatic patients. This is in agreement with another study [13] that stated that effector memory T-cells, CD45RO+, may provide pro-inflammatory signals that contribute to the persistent airway inflammation which is characteristic of asthma, and reduced apoptosis of these cells may prolong their effects.

Surprisingly, there were no significant changes observed in the expression level of CD4 and CD8 markers, nor changes were found in CD4^+^/CD8^+ ^ratio in the studied groups of asthma and HV. This provided evidence that asthmatic inflammation, even though severe, is not solely triggered by CD4^+ ^cells, as previously was believed. This study suggests that asthmatic inflammation originates from both CD4^+ ^and CD8^+ ^cells. This finding is backed up by a previous report [25] which identified the presence of allergen-specific CD8^+ ^T cells. As a remarkable finding, the percentage of CD8^+ ^cells was positively correlated with the percentage of memory cells, CD45RO^+ ^PBL, in severe asthmatics. This finding might indicate an increasing role of the memory cells, CD8^+^CD45RO^+ ^in the pathogenesis of severe asthmatic inflammation.

Spontaneous in vitro synthesis of cytokines was pursued in this study to avoid any induced T helper polarization. Some reports demonstrated an increased PBL production of IFN-γ in subjects with asthma via induced in vitro synthesis [26,27] which is thought to be a biased Th1 polarization as a response to the induction stimuli of in vitro synthesis [28]. ELISA was designed to measure the level of the in vitro synthesis of IL-4 as Th2 prototype cytokine and IFN-γ as Th1 prototype cytokine. The in vitro synthesis of IFN-γ was significantly increasing with asthma severity. It was higher in severe persistent than in mild asthmatics and was higher in mild asthmatics than in HV group. On the other hand, the pattern of the in vitro synthesis of IL-4 was totally different. It was significantly highest in mild asthmatics and low in both severe asthmatics and HV group. Therefore, Th1 cytokines, which were represented by IFN-γ, appeared to take a larger role in asthmatic pathogenesis as disease becomes more chronic and severe. There was a report revealed that the frequencies of T cells producing IFN-γ, but not type 2 cytokines, increased in subjects with asthma compared with normal subjects [29]. However, the role of IFN-γ in causing or aggravating asthma was not proved by other studies [30,31]. It is worth mentioning that Th2 hypothesis of asthma is defined as the consequent alteration in cytokine milieu with excess Th2 products in concert with decreased Th1 products [32]. Therefore, it is paradoxical to relate the increased production of IFN-γ in asthmatics with Th2 hypothesis of asthma given the cross inhibition between IFN-γ and Th2 cytokines [33]. Nevertheless, the findings of this study might solve this confusion. The inflammatory reactions in mild intermittent asthma group were driven mainly by Th2 cytokines because of the very high level of IL-4 and moderately low level of IFN-γ. On the other hand, when asthma became chronically severe, Th1 cytokines became the main player because of the very high level of IFN-γ and low level of IL-4. Accordingly, IL-4/IFN-γ, or Th2/Th1, ratio was significantly lower in severe asthmatics than in both mild asthmatics and HV group. Therefore, PBL of HV group expressed a balanced Th2/Th1 profile with low level of both IL-4 and IFN-γ cytokines. PBL of mild asthmatics expressed balanced Th2/Th1 with mild tendency to Th2, not purely dominant Th2 as previously thought. And PBL of severe asthmatics expressed Th1-polarized profile with very high level of IFN-γ and lower levels of IL-4. Actually this counteracts partially the Th2 hypothesis of asthma that does not discriminate between severe and mild asthma as different entities.

## Conclusion

Taken together, asthma is a disease of different pathogenetic backgrounds. The immune reactions in mild intermittent asthmatics seem to be mediated mainly by Th2 cytokines, with shorter life spans of PBL due to better functioning apoptotic pathways, with less memory cells of PBL, and both CD4^+ ^and CD8^+ ^cells affect the disease without bias to each of them. On the other hand in severe persistent asthma, immune reactions seem to be mediated increasingly by Th1 cytokines, with longer life span of PBL due to progressive loss of apoptosis, with expanded compartment of memory cells in PBL leading to more persistent inflammatory reactions, and both CD4^+ ^and CD8^+ ^cells seem to act together in the disease with predilection to CD8^+ ^type of memory cells, CD45RO^+ ^cells. Collectively, this scenario provides the bases for persistent less allergen-dependent inflammation in severe asthmatic patients. In respect to the current findings, it might be necessary to adjust asthma therapy to the type and severity of asthma where different mechanisms of allergic inflammation and immune responses take place.

## Competing interests

The authors declare that they have no competing interests.

## Authors' contributions

ASA and RRH carried out patients sampling and interviewing, lymphocytes isolation, and did the immunostaining. FA carried out the ELISA for in vitro synthesis of cytokines. KAA and ASA carried out the paper drafting, statistical design, statistical analysis, and the proofreading of the article language and integrity. All authors read and approved the final manuscript.
